# Seizure-Induced Cardiomyopathy: A Case of Takotsubo Cardiomyopathy Following an Epileptic Event

**DOI:** 10.7759/cureus.39288

**Published:** 2023-05-21

**Authors:** Fereshteh Yazdi, Melodie Blackmon, Ayeesha Kattubadi, Prathik Krishnan

**Affiliations:** 1 Internal Medicine, Louisiana State University Health Sciences Center, Shreveport, USA; 2 Critical Care Medicine, Louisiana State University Health Sciences Center, Shreveport, USA; 3 Pulmonary Critical Care, Louisiana State University Health Sciences Center, Shreveport, USA

**Keywords:** critical care cardiology, impella device, alcohol use disorder (aud), epilepsy disorders, takotsubo cardiomyopathy

## Abstract

We present a case, written with the assistance of the Chat Generative Pre-training Transformer (ChatGPT) Artificial Intelligence (AI), of a 75-year-old female with a history of hypertension, epilepsy, coronary artery disease, and alcohol use disorder. She presented with a tonic-clonic seizure, tachycardia, and a cyanotic right hand. Diagnostic tests revealed stress-induced cardiomyopathy, patent bilateral subclavian and axillary arteries with heavy calcification of bilateral upper extremity arteries, and a small filling defect in the segmental branch of the left lower lobe. The patient was started on antiepileptic medication, thiamine/folate, and heparin drip for limb ischemia. Despite treatment with multiple anti-arrhythmic agents, the patient developed cardiogenic shock and underwent left heart catheterization with Impella placement. The Impella was removed 72 hours after placement, and the patient was started on low-dose Milrinone and Levophed for hemodynamic support. The patient eventually recovered and was discharged to long-term acute care.

## Introduction

Stress-induced cardiomyopathy, also known as Takotsubo cardiomyopathy, is a transient cardiac dysfunction that mimics acute coronary syndrome, with similar chest pain characteristics, elevation in cardiac enzymes, and electrocardiogram (EKG) changes [1]. It is often triggered by an acute emotional or physical stressor and is more common in women. The acute decline in heart function is generally resolved in a few days to weeks [2]. Although rare, Takotsubo could be triggered by epilepsy, especially in status-epilepticus. Recent studies suggest that one in every 1000 hospitalizations related to epilepsy might be complicated by Takotsubo cardiomyopathy [3]. In this study, we present a case of a non-compliant epileptic patient who presents to the hospital after a tonic-clonic seizure episode and cardiogenic shock secondary to Takotsubo cardiomyopathy. With the recent development and the integration of artificial intelligence (AI) in scientific writing, this manuscript was written utilizing Chat Generative Pre-training Transformer (ChatGPT) as described below.

## Case presentation

**Figure 1:**
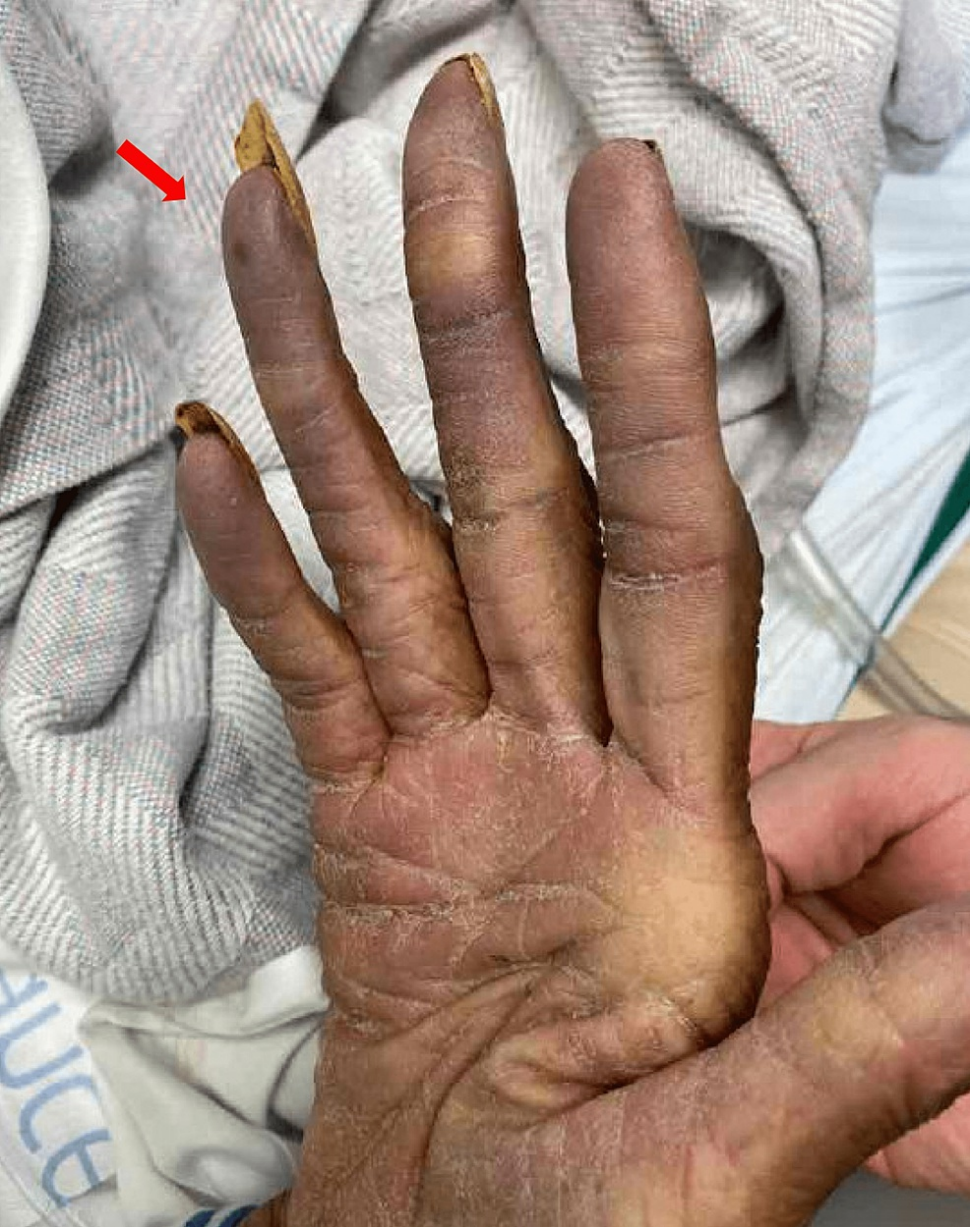
Ischemic right hand with evidence of cyanosis in fingertips.

A 75-year-old female with a medical history of hypertension, epilepsy, and alcohol use disorder, as well as coronary artery disease (CAD) status post percutaneous coronary intervention (PCI) to the obtuse marginal artery (unknown date), presented to the ED with a tonic-clonic seizure, tachycardia, and a cyanotic right hand (Figure 1). Her initial vital signs were significant for a heart rate of 134, blood pressure of 103/51, and oxygen saturation of 97% on room air. Her EKG showed evidence of supraventricular tachycardia that was resolved after a dose of adenosine by the ED. Her high-sensitivity troponin levels were also elevated (836). Due to her hypotension, tachycardia, and post-ictal confusion, she was admitted to the critical care unit for closer monitoring.

**Figure 2:**
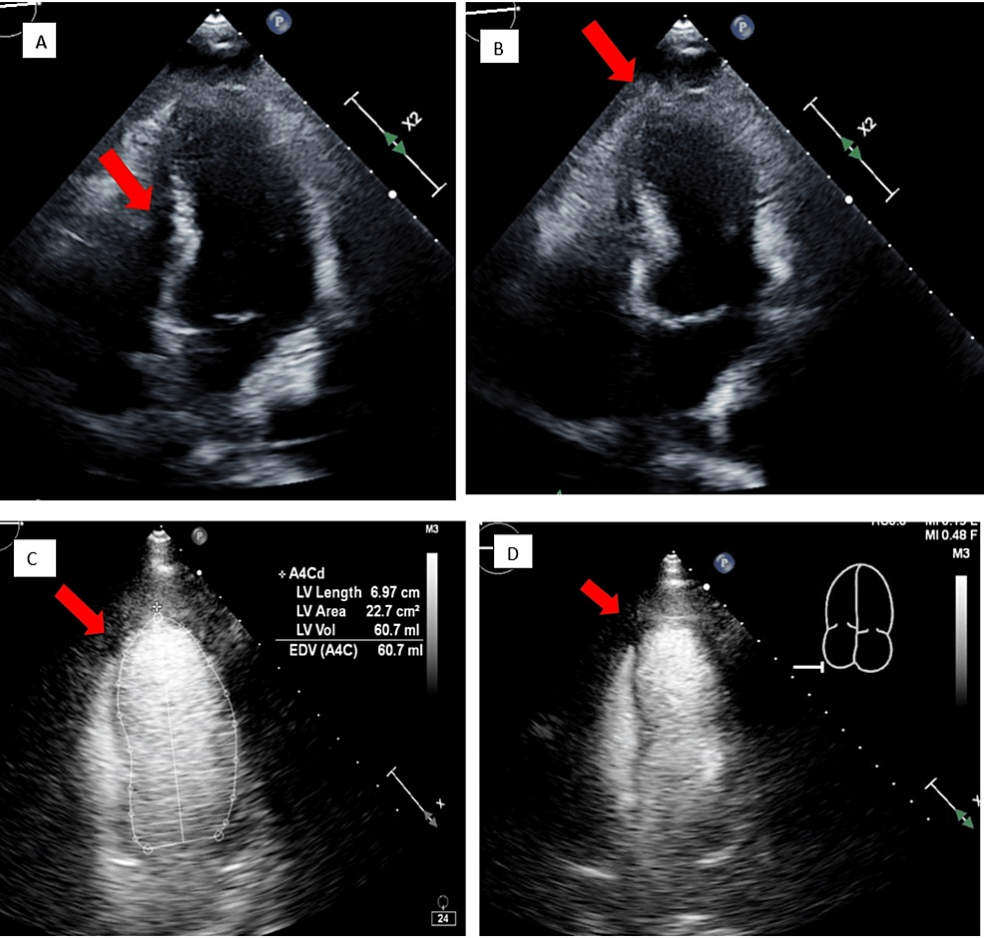
Echocardiogram images of hypokinetic mid-apical segments with preserved contractility of basal segments (Takotsubo cardiomyopathy) during systole and diastole. Figures A and B are without contrast, and Figures C and D are with contrast images.

**Figure 3:**
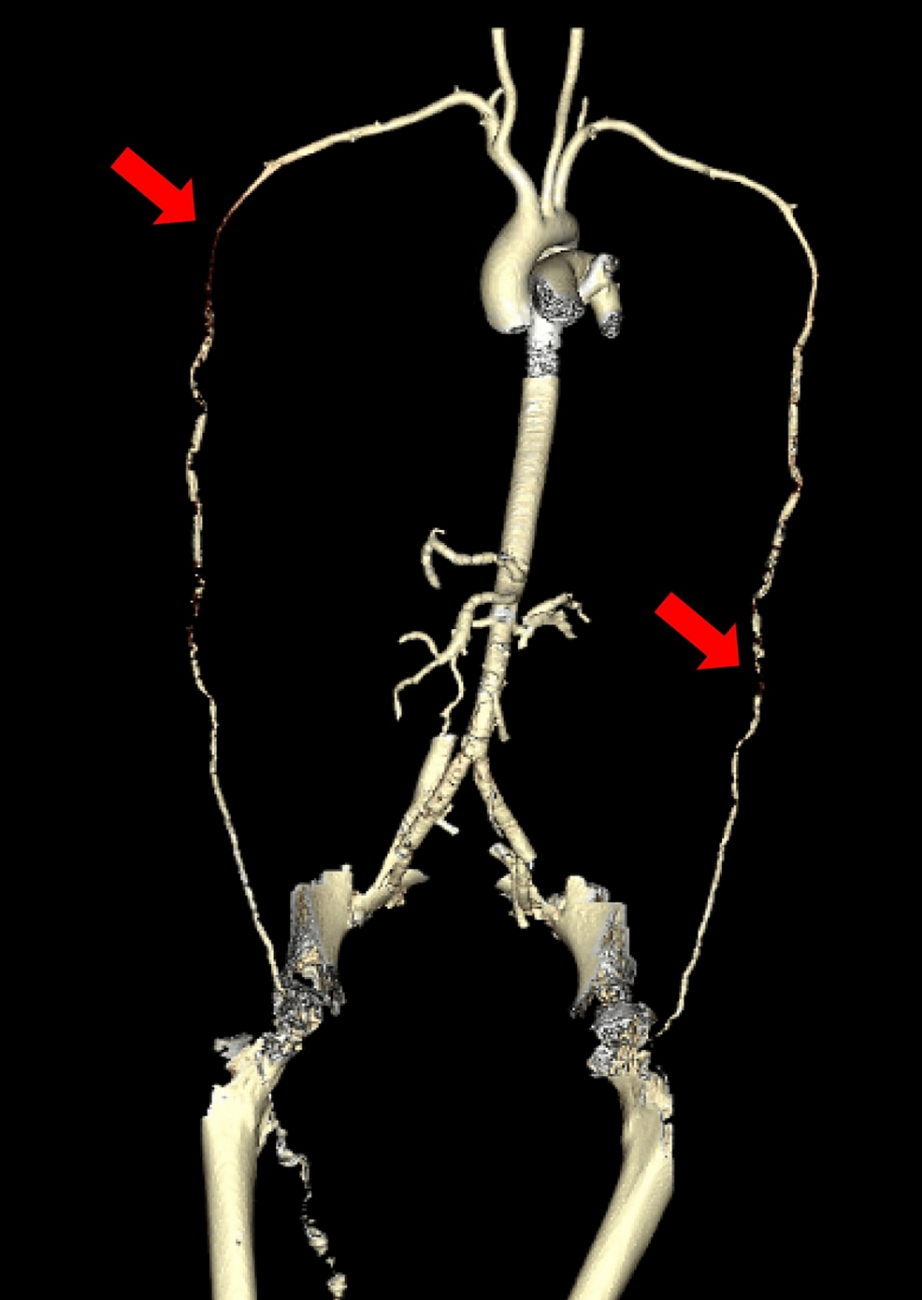
Computed tomography angiography of the chest and upper extremities with evidence of patent bilateral subclavian and axillary arteries with heavy calcification of upper extremity arteries distal to this point.

The patient denied any pain or claudication of her right hand on the initial presentation, and the family was unable to provide further history regarding the timeline or associated symptoms with her hand ischemia. Initial diagnostic tests, such as an echocardiogram, revealed severely reduced ejection fraction (15-20%) and severely hypokinetic mid-apical segments (Figure 2) with preserved contractility of basal segments, consistent with stress-induced cardiomyopathy. No prior echocardiograms were available for comparison studies. An Angiogram of the chest and upper extremities revealed patent bilateral subclavian and axillary arteries, heavy calcification of bilateral upper extremity arteries (Figure 3), and a small filling defect in the segmental branch of the left lower lobe.

**Figure 4:**
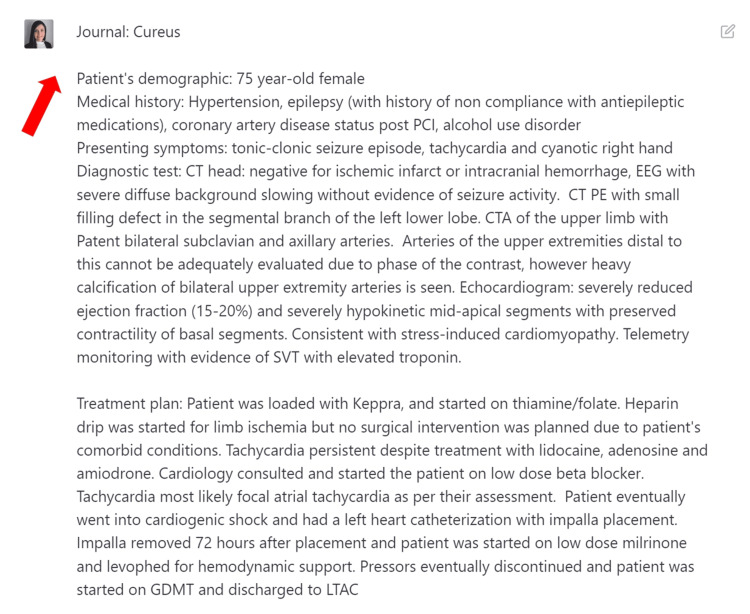
The information input for ChatGPT to generate the case report. ChatGPT: Chat Generative Pre-training Transformer.

**Figure 5:**
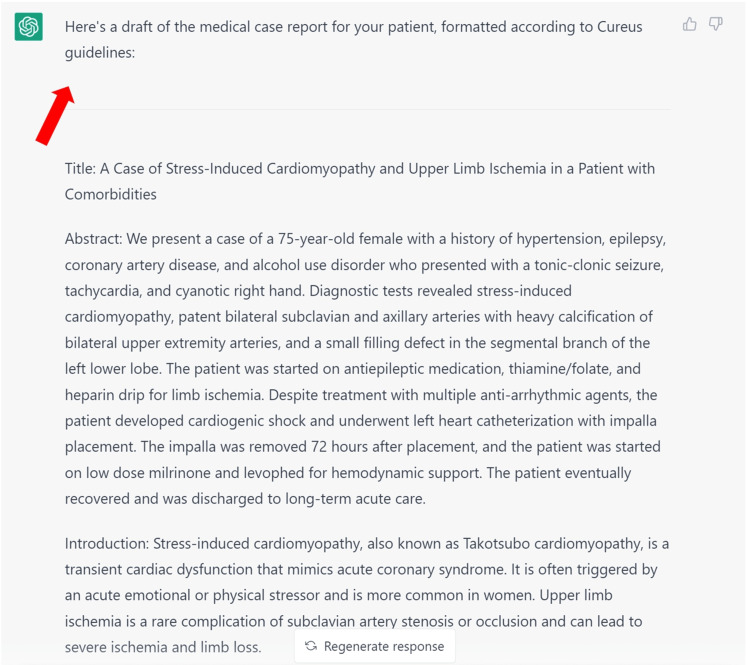
The title, abstract, and introduction output by ChatGPT as per Cureus Journal guidelines. ChatGPT: Chat Generative Pre-training Transformer.

**Figure 6:**
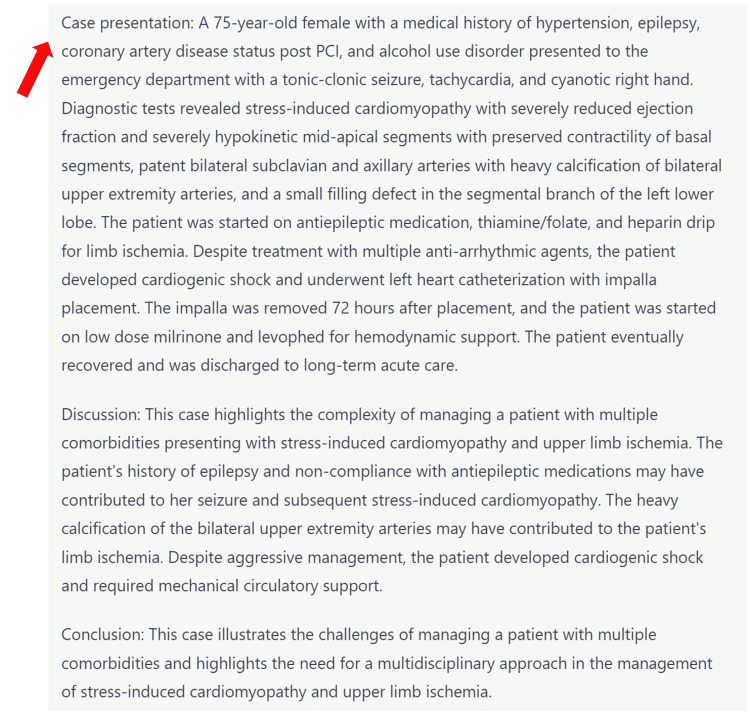
The case presentation, discussion, and conclusion generated by ChatGPT prior to the authors' edits and formatting. ChatGPT: Chat Generative Pre-training Transformer.

The patient was started on antiepileptic medication, thiamine/folate, and heparin drip for limb ischemia. Vascular surgery evaluated the patient, but no intervention was deemed necessary for limb ischemia. The etiology of her ischemia and the timeline of when her discoloration started remains unknown. Despite treatment with vasopressors and inotropes, the patient developed cardiogenic shock, with the ejection fraction reducing to 10%. She subsequently underwent left heart catheterization with Impella pump placement for cardiopulmonary support. No obstructive CAD was found during catheterization, and only mild in-stent restenosis was noted on her prior PCI. The Impella was removed 72 hours after placement, and the patient was continued on low-dose Milrinone and Levophed to ensure adequate blood pressure and cardiac contractility. The patient eventually recovered and was discharged to long-term acute care. We utilized AI in drafting this manuscript, specifically ChatGPT. Our correspondence with the AI is depicted in Figures 4-6. 

## Discussion

We present a rare presentation of Takotsubo cardiomyopathy induced by a seizure episode in a non-compliant patient. Her presentation of tachycardia (SVT) and elevated troponin was initially concerning for acute coronary artery syndrome, especially with concurrent limb ischemia and pulmonary embolism. However, she was found to have only mild CAD during her left heart catheterization. Her echocardiogram also showed no evidence of dilated cardiomyopathy that would suggest her cardiogenic shock and reduced ejection fraction were secondary to alcohol abuse. This case highlights the importance of including stress-induced cardiomyopathy as a differential diagnosis in a patient with epilepsy and poor cardiac function, as timely management can improve mortality rates [4]. Epilepsy is a known cause of Takotsubo cardiomyopathy, and cardiac complications are the main cause of mortality in epileptic patients [5]. It has been postulated that the rise in catecholamines during a stressful situation, such as a seizure episode, as well as vasospasm or microvascular failure leads to increased cardiac demand and, eventually, cardiac failure [6]. Takotsubo cardiomyopathy after seizures has been suspected as a cause of sudden unexplained death in epilepsy (SUDEP) in some studies [7]. As a result, timely diagnostic approaches such as echocardiograms should be prioritized in elderly patients or those with prolonged seizure episodes. We also utilized ChatGPT to assist with this case report's initial write-up. We acknowledge the advances in technology and the shift in scientific writing. We demonstrate the information input from our patient into ChatGPT and showcase the results of the case described by AI. The text of this case report was then edited to include scientific references and ensure the accuracy of the content prior to submission for publication. 

## Conclusions

This case illustrates the challenges of managing a patient with multiple comorbidities and highlights the need for a multidisciplinary approach to managing stress-induced cardiomyopathy. There should be a low threshold for investigating cardiac function in high-risk patients following an epileptic episode.
